# Detection of Streptavidin Based on Terminal Protection and Cationic Conjugated Polymer-Mediated Fluorescence Resonance Energy Transfer

**DOI:** 10.3390/polym13050725

**Published:** 2021-02-27

**Authors:** Tingting Hu, Ying Yan, Zhenwei Tang, Xinfa Liu, Changbei Ma

**Affiliations:** School of Life Sciences, Central South University, Changsha 410013, China; hutingting516@163.com (T.H.); Yany2018@csu.edu.cn (Y.Y.); 2204140108@csu.edu.cn (Z.T.)

**Keywords:** streptavidin, FRET, terminal protection, conjugated polymer

## Abstract

In this paper, a fast and simple strategy for sensitive detection of streptavidin (SA) was proposed based on terminal protection of small molecule-linked DNA and cationic conjugated polymer-mediated fluorescence resonance energy transfer (FRET). In principle, we designed a biotin-labelled DNA probe (P1) as the recognitive probe of SA, along with a complementary DNA probe (P2) to form double-stranded DNA (dsDNA) with P1. SYBR Green I (SG I) as a fluorescent dye was further used to specifically bind to dsDNA to emit stronger fluorescence. The cationic poly[(9,9-bis(6′-N,N,N-triethy-lammonium)hexyl) fluorenylene phenylene dibromide] (PFP) acted as the donor to participate in the FRET and transfer energy to the recipient SG I. In the absence of SA, P1 could not hybridize with P2 to form dsDNA and was digested by exonuclease I (Exo I); thus, only a weak FRET signal would be observed. In the presence of SA, biotin could specifically bind to SA, which protected P1 from Exo I cleavage. Then, P1 and P2 were hybridized into dsDNA. Therefore, the addition of SG I and PFP led to obvious FRET signal due to strong electrostatic interactions. Then, SA can be quantitatively detected by monitoring FRET changes. As the whole reagent reaction was carried out in 1.5 mL EP and detected in the colorimetric dish, the operation process of the detection system was relatively simple. The response time for each step was also relatively short. In this detection system, the linear equation was obtained for SA from 0.1 to 20 nM with a low detection limit of 0.068 nM (S/N = 3). In addition, this strategy has also achieved satisfactory results in the application of biological samples, which reveals the application prospect of this method in the future.

## 1. Introduction

Streptavidin (SA) is a 58.2 kDa protein secreted by the bacterium *Streptomyces avidinii* and composed of four identical peptide chains contains tryptophan, which can bind to biotin with high specificity and strong affinity [1,2]. The combination of SA and biotin, one of the strongest non-covalent effects known in nature, has been a research hotspot with great significance and prospect [3]. Furthermore, the streptavidin–biotin complex play a role in biological anode technology and molecular biology due to their excellent biological tolerance to extreme conditions such as proteolytic enzymes, detergents (e.g., SDS), denaturators (e.g., guanidinium chloride), and extremes of temperature and pH [4,5]. Besides, because of the specific interactions of SA and biotin, the development of a highly sensitive SA detection strategy is very beneficial for applications in disease prediction, chemical genetics, and drug development [6].

At present, an increasing number of methods have been developed for SA detection and quantitative analysis, including protein-fragment complementation assay [7], affinity chromatography [8], kinetic capillary electrophoresis [9] and surface plasmon resonance [7,10]. However, most of these traditional methods have disadvantages such as small samples, expensive instruments, time-consuming detection and cumbersome operation processes, which greatly limit their application [11]. Therefore, it is urgent to obtain a fast, low-cost and highly sensitive detection approach for the quantitative analysis of SA. 

SYBR Green I (SG I) is a dye with Green excitation wavelength that can specifically bind to double-stranded DNA (dsDNA) double helix groove region [12,13]. In the free state, SG I fluoresces weakly, but when it binds to dsDNA, the fluorescence is greatly enhanced. This phenomenon can be used to detect dsDNA based on the strength of the fluorescence signal [14,15], with a maximum absorption wavelength at about 497 nm, and the maximum emission wavelength at about 520 nm. On the other hand, PFP is a novel kind of water-soluble cationic conjugate polymer with positive charge, which can adsorb dsDNA through strong electrostatic interaction [16]. When SG I is mixed with PFP, the PFP can occur fluorescence resonance energy transfer (FRET) by interacting with SG I embedded in the double-stranded structure [17]. Therefore, quantitative detection can be achieved according to the efficiency changes of FRET.

Recently, the special interaction between proteins and small molecules has attracted attention due to its wide application value in molecular diagnosis, anticancer treatment, drug development and other areas [4,18,19]. In addition, the research on the specific binding of small molecules to proteins not only builds understanding on the mechanism of small molecules and improve the detection methods of proteins, but also has important significance in the fields of biochemistry and clinical medicine [20,21]. Currently, studies have reported that the terminal modified DNA resistance to degradation by the 3’ single-strand-specific exonuclease I (Exo I) by virtue of the tight connection is called the terminal protection of small molecule-linked DNA [22,23,24]. Hence, this may be an ideal choice for protein detection.

Herein, based on terminal protection-mediated FRET, an amplification method was developed for the detection of SA. We designed a biotin-labelled DNA sequence called P1 as the substrate of SA and a complementary hybrid DNA sequence called P2. In the presence of SA, biotin can specifically bind to SA, which protects P1 from Exo I cleavage. Then, P1 and P2 were hybridized into dsDNA. Therefore, the addition of SG I and PFP led to obvious FRET signal due to strong electrostatic interactions. On the contrary, in the absence of SA, P1 was cleaved by Exo I and cannot hybridize with P2, which prevents the formation of dsDNA, so only weak FRET signal can be observed. As a result, SA can be quantitatively detected by monitoring FRET changes. Furthermore, the proposed method showed a high sensitivity and selectivity for SA detection.

## 2. Materials and Methods

### 2.1. Materials and Measurements

The DNA sequences are as follows. P1: 5′-CGACATCTAACCTAGCTGACT-3′ P2: 5′-AGTCAGCTAGGTTAGATGTCG-3′. The two oligonucleotides, P1 and P2, were purchased and purified from Sangon Biological Engineering Technology & Services Co., Ltd. (Shanghai, China). Streptavidin (SA), Carcinoembryonic antigen (CEA), alpha fetoprotein (AFP) and Immunoglobulin G (IgG) were all obtained from Sigma-Aldrich (St. Louis, MO, USA). The ultrapure water (18.2 MΩ.cm) used in the experiments was from a Milli-Q water purification system (Millipore Corp, Bedford, MA, USA). The reaction buffer in the experiment system was Tris-HCl buffer (20 mM Tris-HCl, 150 mM NaCl, 10 mM MgCl_2_, pH7.5). Tris [Tris- (hydroxy- methyl) aminomethane], hydrochloric acid (HCl), sodium chloride (NaCl) and agnesium chloride (MgCl_2_) were purchased from Sinopharm Chemical Reagent Co., Ltd. (Shanghai, China). Exonuclease I were obtained from New England Biolabs (Beverly, MA, USA). SYBR green I (20×) was purchased from ZeesanBiotech co., Ltd. (Xiamen, China). Poly[(9,9-bis(6′-N,N,N-triethy-lammonium)hexyl) fluorenylene phenylene dibromide] (PFP) was purchased from Yuanye Co., Ltd. (Shanghai, China). 

### 2.2. Apparatus

An F-2700 fluorescence spectrophotometer (Hitachi, Japan) was used to record the fluorescence measurement with both excitation and emission slit at 10.0 nm. The photomultiplier tube voltage was 700 V. The emission spectra were set in a range of 400–600 nm when the excitation wavelength was set as 380 nm for the PFP. Each experiment was carried out in a final volume of 100 µL. The result error was obtained from three repeated measurements, and statistical methods were used to collate and analyze the data during the experiment.

### 2.3. Method Optimization

In order to obtain the best detection system, several important conditions were optimized, such as the concentration of P1, Exo I, P2, PFP, and the reaction time of SA and P1. The concentrations range of P1, Exo I, P2, and PFP were 50–300 nM, 0–40 U/mL, 50–300 nM, and 0.8–1.6 μM, respectively. The reaction time range of SA and P1 was 0–100 min. The final volume of the reaction system was set at 100 μL, and each experiment was repeated three times.

### 2.4. Detection of SA

The following method was performed to quantitatively detect SA. Firstly, for the binding of SA to biotin labeled at the end of P1, SA with various concentrations ranging from 0 to 100 nM and 200 nM P1 were added the reaction buffer (20 mM Tris-HCl, 150 mM NaCl, 10 mM MgCl_2_, pH 7.5) at 37 °C for 60 min. Secondly, 15 U/mL Exo I was added the above mixed solution and incubated for 30 min at 37 °C. Subsequently, Exo I was added at 90 °C for 10 min. Then, 200 nM P2 was added to the solution and hybridized with P1 to form dsDNA. Finally, SG I and 1.2 μM PFP were added and the mixture was stored in the dark at room temperature for 20 min before carrying out the F-2700 fluorescence spectrophotometer. To evaluate the practical application of this assay, we detected different concentrations of SA in diluted serum.

## 3. Results

### 3.1. Experimental Principles

**Scheme 1:**
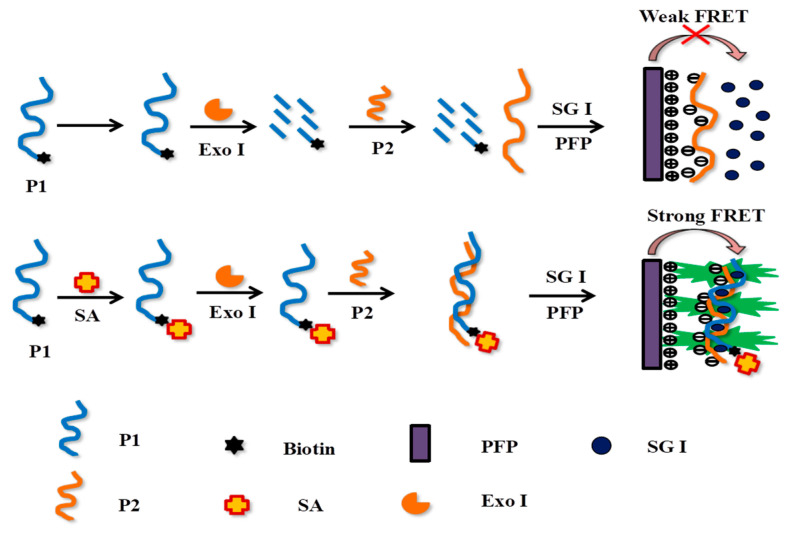
Working principal of the SA detective strategy based on terminal protection-mediated fluorescence resonance energy transfer amplification.

The mechanism of SA detection proposed in this paper was illustrated in Scheme 1. As is shown, a biotin-labelled DNA sequence called P1 (the blue) as the substrate of SA and a complementary hybrid DNA sequence called P2 (the orange) were designed. In the presence of SA, biotin labeled at the end of P1 can specifically bind to SA, which protects P1 from Exo I cleavage [3]. Then, the DNA strand P1 can be hybridized with P2 to form dsDNA. Subsequently, the added SG I bond to the dsDNA to produce high fluorescence. When the PFP was added, due to strong electrostatic interaction, the dsDNA was moved in close proximity to the PFP [25]. As a result, the efficient FRET from PFP to SG I was occurred and an amplified fluorescence signal was detected. However, in the absence of SA, the DNA strand P1 was cleaved by Exo I and cannot hybridize with P2, which prevents the formation of dsDNA, so only a weak FRET signal can be detected. Accordingly, the amount of SA can be quantitatively determined by monitoring FRET changes.

### 3.2. Feasibility Assessment of the SA Detection Assay

**Figure 1:**
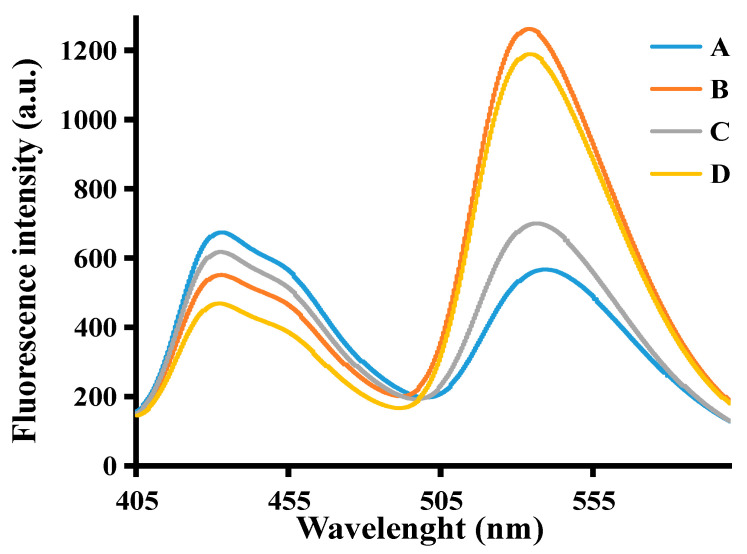
The feasibility of the proposed method. (A) P1 + SG I+ PFP; (B) P1 + P2 + SG I+ PFP; (C) P1 + Exo I+ P2 + SG I + PFP; and (D) P1 + SA + Exo I + P2 + SG I + PFP. Concentrations of P1, SA, Exo I, P2, SG I and PFP were 150 nM, 150 nM, 25 U/mL, 150 nM, 1 X and 0.8 μM, respectively.

To verify the feasibility of this proposed approach, we measured the fluorescence emission spectra of the SA detection system under different conditions. As shown in Figure 1, when only the P1 was added, PFP exhibited high fluorescence at 430 nm and SG I showed low fluorescence at 535 nm because SG I could not bind with the ssDNA (curve A). When the P1 and P2 were added, the fluorescence decreased at 430 nm and increased at 535 nm, which was caused by the FRET because of the formation of dsDNA between P1 and P2 (curve B). However, when Exo I was added before P2, it can be observed that the FRET ratio (I_535_ nm/I_430_ nm) was significantly reduced, indicating that the digestion by Exo I to P1(curve C). In contrast, when SA was added into the mixture, the FRET ratio (I_535_ nm/I_430_ nm) increased dramatically again, owing to protection of SA to P1 and the formation of dsDNA (curve D). These results implied that the detection system has good feasibility and could carry out the following experiments.

### 3.3. Optimization of Experimental Conditions

**Figure 2:**
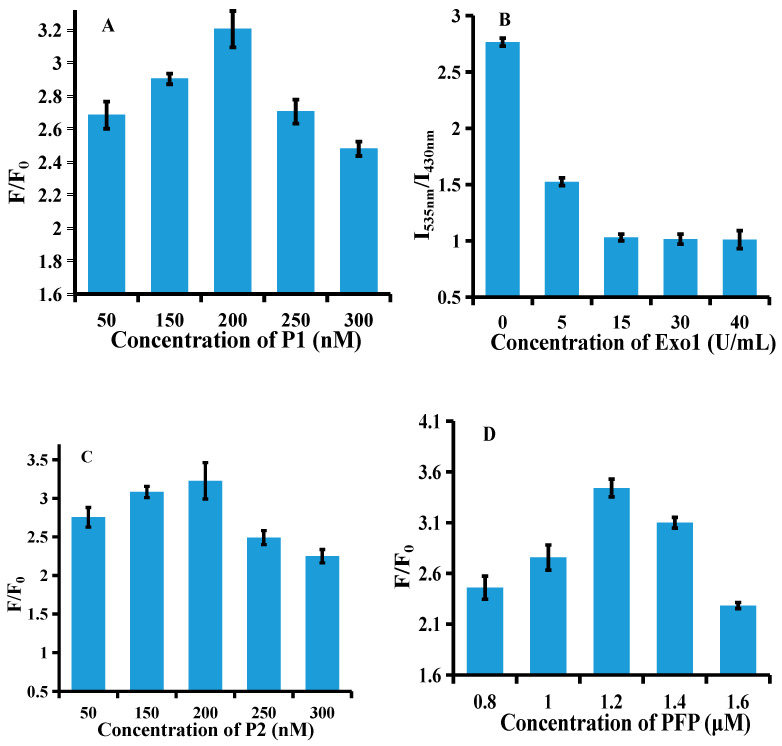

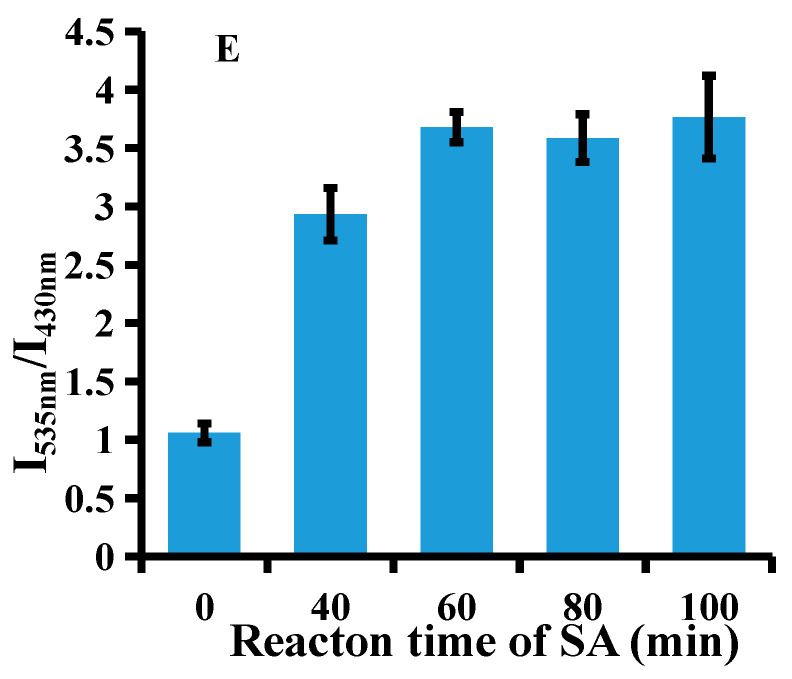
Optimization of experimental conditions. (**A**) concentrations of P1 (50, 150, 200, 250, 300 nM); (**B**) concentrations of Exo I (0, 5, 15, 30, 40 U/mL); (**C**) concentration of P2 (50, 150, 200, 250, 300 nM); (**D**) concentrations of PFP (0.8, 1, 1.2, 1.4, 1.6 μM); (**E**) SA reaction time (0, 40, 60, 80, 100 min).

As shown in Figure 2, to obtain the best reaction system, several conditions which could have great influence on the reaction system were optimized. In each optimization experiment, the concentration of other conditions remains unchanged. It was worth noting that the reaction temperature of SA was a very important factor in this detection system. However, through simple optimization of pre-experiment and combined with relevant references, we finally chose 37 °C as the temperature of SA reaction and applied it in the experiment [2]. Two parameters were included to evaluate the signal. I_535_ nm/I_430_ nm, which refers to the ratio of the fluorescence intensity at 535 nm to 430 nm. F/F_0_ refers to the ratio of the I_535_ nm/I_430_ nm in the presence and absence of SA. Firstly, P1 with a concentration range of 50–300 nm was optimized, as shown in Figure 2A, 200 nm was the optimal concentration of P1. 

As is indicated in Figure 2B, we explored the effect of Exo1 at concentrations of 0–40 U/mL on the experimental system by measuring the ratio of the I_535_ nm/I_430_ nm and selected 15 U/mL for the next experiment. Next, the concentrations of P2 between 50 and 300 nm were optimized. As can be seen in Figure 2C, the F/F_0_ was highest at the concentration of P2 at 200 nm, so 200 nm P2 was set as the optimal concentration. Then, we investigated the optimal concentrations of PFP. It can be illustrated from Figure 2D that 1.2 μM was the optimal reaction concentration of PFP. Finally, we explored the optimal incubation time for the effect of SA and P1. As shown in Figure 2E, after the reaction time of SA and P1 reached 60 min, the I_535_ nm/I_430_ nm hardly changed. Therefore, 60 min was selected in subsequent experiments.

### 3.4. Quantitative Detection of SA 

**Figure 3:**
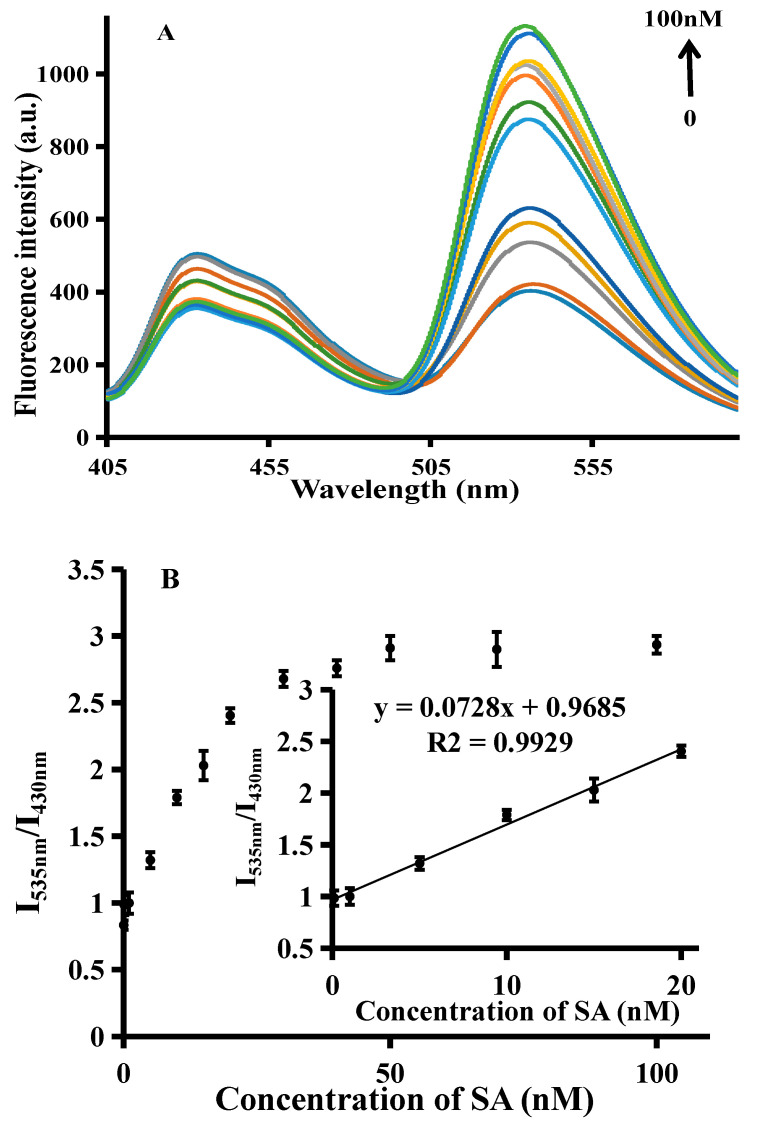
(**A**) Fluorescence emission spectra in the presence of increasing amounts of SA (0, 0.1, 1, 5, 10, 15, 20, 30, 40, 50, 70, 100 nM). (**B**) Graph depicting the changes in fluorescence output at 430 nm (PFP) and 535 nm (SG I) as a function of SA concentration. Inset: Linear relationship between fluorescence intensity and low SA concentrations. Error bars were estimated from three replicate measurements.

Under the above optimal reaction conditions, we selected the concentration range of SA from 0 to 100 nM (0, 0.1, 1, 5, 10, 15, 20, 30, 40, 50, 70, 100 nM) for quantitative detection of SA. Figure 3A shows the fluorescence emission spectra of SA with different concentrations under the optimal detection system. It can be seen that the I_535_ nm/I_430_ nm increased gradually with the increase of SA concentration. As seen in Figure 3B, the relationship between the I_535_ nm/I_430_ nm and the concentrations of SA can be observed. As shown in the inset of Figure 3B, there was a good linear correlation (R^2^ = 0.9929) between the I_535_ nm/I_430_ nm and SA concentration range of 0 to 20 nM. Moreover, the linear regression equation of Y = 0.0728X + 0.9685 was obtained, where X is the concentration of SA (nM) and F is the value of I_535_ nm/I_430_ nm. According to the 3σ rule, the limit of detection (LOD) was estimated to be 0.068 nM. For example, Huang et al. reported a paper-based electrochemiluminescence method based on reticulated DNA functionalized PTCU nanoframes and analysis of triggered DNA Walker for SA determination [26]. Due to the use of chain substitution cycle amplification strategy, the detection limit of this method is lower than our method. However, because of the use of electrochemiluminescence detection, this method is more complex than the method in this paper, and the cost is also higher. In addition, compared with other most methods for SA detection in recent years, the detection limit in this paper is comparable to or lower than that of most methods mentioned above (Table 1).

### 3.5. Selectivity of SA Assay

**Figure 4:**
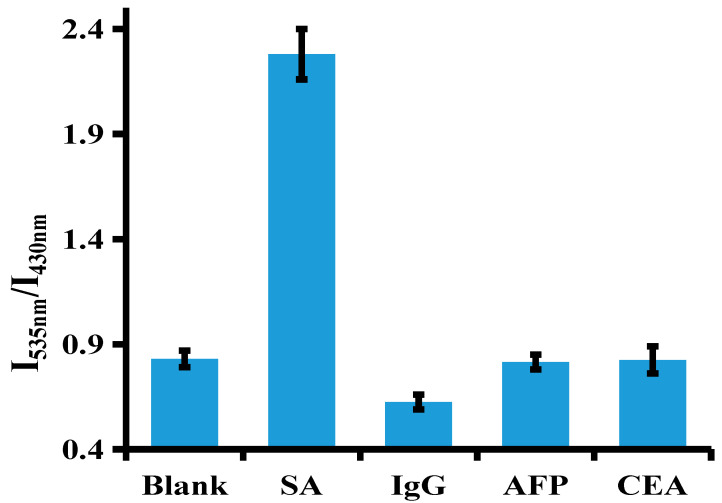
Fluorescence intensity in the presence of SA, IgG, AFP, and CEA. Error bars were estimated from three replicate measurements.

In order to further test the specificity of the SA detection system, 20 nM of interfering proteins, such as IgG, AFP, CEA, SA and blank were selected for detection under optimized experimental conditions. As displayed in Figure 4, there was a significant increase in I_535_ nm/I_430_ nm ratio with only SA, while the FRET changes triggered by IgG, AFP, and CEA were nearly negligible. This result was mainly attributed to the specific interaction between SA and biotin, which indicated that the detection method in this paper has good selectivity.

### 3.6. Application of the Proposed Assay in Biological Systems

To further evaluate the application value of this method in biological samples, we prepared 100-fold diluted human serum sample to detect SA. Then, we selected three different concentrations of SA (5, 10, 14 nM) to add to the diluted serum, and the SA concentrations were measured by the sensor platform proposed above. As seen in Table 2, we obtained the recovery rates of SA at different concentrations in diluted serum, such as 97.5% for 5 nM, 103.9% for 8 nM, and 90.5% for 15 nM, and the RSD were 9.9%, 6.88%, and 3.25%, respectively. Therefore, the results indicated that the proposed strategy could be potential for SA detection in biological systems.

## 4. Conclusions

The SA–biotin system composed of SA and biotin can bind to antigens, antibodies, enzymes, oligonucleotide molecules and fluorescent substances, and has a wide range of applications in biology, especially in the detection of immune detection, antigen, antibody and nucleic acid molecules. However, the development of these detection methods also faces great challenges, and the detection method based on FRET in this paper has high experimental requirements. If the spectrum overlap between donor and recipient is not good, it will lead to fluorescence interference, and it is difficult to observe instantaneous molecular interactions, which requires a large number of samples. Currently, the SA–biotin system is used in most of the various detection kits based on the principles of enzyme-linked immune response, fluorescent molecular labeled immune response and molecular hybridization. However, all the SA used in China are expensive imported products, so it is of great practical significance and broad application prospect to develop a domestic SA and find a highly sensitive SA detection method.

In conclusion, a simple, sensitive and specific method for the SA detection based on terminal protection-mediated fluorescence resonance energy transfer amplification was successfully established. Due to the application of the FRET amplification strategy in the method, a low detection limit of 0.068 nM (S/N = 3) was obtained, and the linear range was 0.1 to 20 nM under the optimized conditions. Compared with most other fluorescence methods for SA detection, we obtained a lower detection limit and a wider linear range, which indicated that our method was more sensitive and valuable for research. Furthermore, the SA detection system has achieved satisfactory results in both selectivity assays and the biological sample tests. The recoveries of the three samples were in the range of 90%–110%, and the RSD was less than 10%. Therefore, it can be inferred that this method proposed in this paper has great application prospects in the future development of sensor platforms for SA detection.

## Figures and Tables

**Table 1:** Comparison of different methods for the detection of SA.

Material	LOD (nM)	Dynamic Range (nM)	Reference
CuNP	0.1	0.5–1000	3
FAM	0.08	0.15–12	1
Copper nanocluster	0.47	1–200	2
Gold nanoparticle	5	5–50	7
SYBR Green I	0.016	0.01–0.1	18
SYBR Green I	0.4	0.4–200	4
SYBR Green I	0.068	0.1–20	This work

**Table 2:** Recovery experiments of SA in human serum samples (n = 3).

Sample	Added (nM)	Found (nM)	Recovery (%)	RSD (%)
1	5	4.87 ± 0.51	97.48	9.9
2	8	8.31 ± 0.63	103.85	6.88
3	15	13.57 ± 0.50	90.49	3.25

## Data Availability

The data presented in this study are available on request from the corresponding author. The data are not publicly available due to participant confidentiality.
